# Sports-related wrist and hand injuries: a review

**DOI:** 10.1186/s13018-016-0432-8

**Published:** 2016-09-16

**Authors:** Daniel M. Avery, Craig M. Rodner, Cory M. Edgar

**Affiliations:** University of Connecticut Musculoskeletal Institute, 263 Farmington Avenue, Farmington, CT 06030-4037 USA

**Keywords:** Hand injuries, Wrist injuries, Sports, Return to play, Ligament, Fracture, Surgical treatment

## Abstract

**Background:**

Hand and wrist injuries are common during athletics and can have a significant impact especially if initially disregarded. Due to their high level of physical demand, athletes represent a unique subset of the population.

**Main body:**

The following is an overview of hand and wrist injuries commonly seen in athletics. Information regarding evaluation, diagnosis, conservative measures, and surgical treatment are provided.

**Conclusion:**

Knowledge of these entities and special consideration for the athlete can help the team physician effectively treat these players and help them achieve their goals.

## Background

Approximately 25 % of all sports-related injuries involve the hand or wrist [1, 2], and incidence is growing not only due to the competitive level of high school and collegiate athletes but also due to the activity level of the general population [3]. While the shoulder and knee are the commonly thought of in athletic injury, hand and wrist injuries are common and can have a significant impact especially if initially disregarded with a resultant delay to treatment.

Due to the high level of physical demand for function, athletes represent a unique subset of the population. Injury time can have a significant impact on scholarship opportunities or jeopardize professional aspirations with direct financial impact. Knowledge of common sports-related injuries and therapeutic strategies can help the physician effectively treat the athlete considering their sports, position, and timing during season. The following is an overview of hand and wrist injuries commonly seen in athletics. Information regarding evaluation, diagnosis, conservative measures, and surgical treatment are provided.

### Radial-sided wrist injuries

#### Scaphoid fracture

Scaphoid fractures are the most commonly injured carpal bone [4] with a high incidence in college football players [5] and an increasing incidence in female athletes [6]. This hyperextension wrist injury tends to occur in a pronated, radially deviated hand. Presentation can range from disabling wrist pain to mild swelling and decreased range of motion. It is not uncommon to find a scaphoid nonunion with a remote history of a wrist sprain.

Located at the radial side of the carpus, athletes will complain of radial-sided wrist pain with exquisite tenderness in the anatomical snuff box, axial loading of the thumb, or pincer grasp. Radiographic assessment of the wrist should include a posteroanterior (PA), lateral, and ulnar deviated view. Unfortunately, due to subtle fracture lines and the irregular contour of the scaphoid, nondisplaced fractures can be missed on radiographs and advanced imaging with computed tomography (CT) scan for fracture identification or alignment. Additionally, magnetic resonance imaging (MRI) or bone scintigraphy for occult fracture may be needed to confirm the diagnosis [7, 8].

**Fig. 1:**
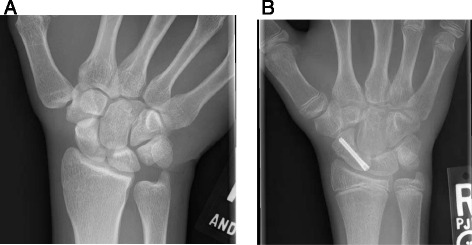
**a** PA radiograph of a nondisplaced proximal pole scaphoid fracture in a recreational hockey player. **b** PA radiograph of a nondisplaced scaphoid waist fracture in a high school soccer player treated with headless compression screw fixation

Treatment decisions depend upon fracture location and displacement, with strong surgical consideration being given to scaphoid fractures which are displaced and/or proximal. Whether treatment affects the athlete’s continued participation in his or her sports within the context of the status of the season may also play a role in determining whether or not to operate. Due to retrograde blood supply, distal pole scaphoid fractures can effectively be treated nonsurgically. Proximal pole fractures are prone to avascular necrosis and necessitate stronger surgical consideration [9, 10]. Likewise, displacement carries a relatively increased risk of nonunion and we would recommend surgical fixation. Operative management, mostly commonly in the form of headless compression screw fixation, often offers the fastest return to sports [11]. Cast immobilization may provide appropriate definitive treatment in a nondisplaced fracture or a temporizing measure for return to play. Return to athletic participation is based upon the athlete’s handedness, his or her specific sports’ requirements, and negotiating the bulk or restriction of the cast with respect to dexterity and/or strength [12] (Fig. 1).

#### Scapholunate ligament tears

Wrist instability commonly occurs in a spectrum of severity in hyperextension injuries. Contact sports such as football or rugby commonly place the athlete in a position of impact with hyperextension, ulnar deviation, and supination of the wrist that can lead to these injuries.

**Fig. 2:**
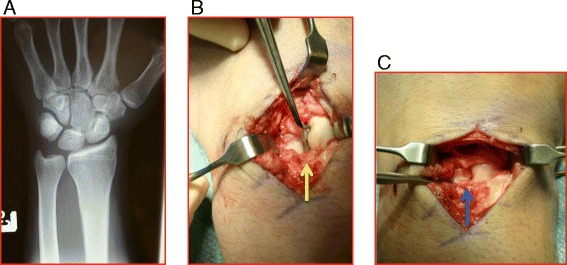
**a** PA radiograph showing a flexed scaphoid (*signet ring sign*). **b** Intraoperative finding of a complete SL interosseous ligament tear with the tip of the probe on the scaphoid (*yellow arrow*). **c** Open reduction of the SL interval (*blue arrow*) prior to ligament repair

Because of the proximity of structures in the wrist, diagnosis of these injuries can be challenging. Pain in a loaded, extended wrist with tenderness in the dorsal wrist at the interval between the third and fourth extensor compartments suggests possible scapholunate (SL) interosseous ligament injury. Standard radiographic assessment with PA and lateral views may appear normal only showing increased flexion of the scaphoid (a signet ring sign on the PA view as in Fig. 2a). A PA clenched fist view may show greater than 5 mm of widening between the scaphoid and lunate (Terry Thomas sign) is diagnostic of a complete SL ligament tear [13]. Chronic tears may demonstrate a static SL gap on the PA film and an increased SL angle on the lateral consistent with dorsal intercalated segmental instability (DISI). Advanced imaging is commonly needed in the form of MRI with or without contrast arthrography [14].

Suspected tears or partial tears can respond to immobilization allowing the participant to still compete. Those with continued pain and dysfunction that interferes with their level of play will require wrist arthroscopy. Geissler et. al. [15] developed an arthroscopic grading system which helps guide management that ranges from immobilization for attenuation of an intact ligament to open reduction and repair for gross instability.

#### Radial-sided tendinopathies

Radial-sided wrist pain from overuse injuries requires careful evaluation. Accurate diagnosis using provocative maneuvers and identifying the precise location of maximal tenderness are paramount. Radiographic assessment can be indicated for ruling out fracture depending on the patient’s history. Advanced imaging, such as MRI, is not routinely used.

The most common tendinopathy in the athlete is de Quervain’s tenosynovitis [16]. Repetitive thumb extension and abduction can lead to a thickening of the abductor pollicis longus and extensor pollicis brevis tendons as they pass under the first extensor compartment retinaculum. Tenderness to palpation is approximately 2 cm proximal to the radial styloid and exacerbated by tucking the thumb under the other fingers while ulnarly deviating the wrist (a positive Finkelstein’s sign) [17, 18].

Intersection syndrome, also called Oarsman’s wrist, is caused by friction at the crossing of the tendons of the first extensor compartment as they pass over the tendons of the second extensor compartment (extensor carpi radialis longus and brevis) or a stenosing tenosynovitis within the second extensor compartment itself [19]. Pain is elicited with extension and radial deviation approximately 4–8 cm proximal to the radial styloid. Without careful attention to the location of pain, this can be misdiagnosed as de Quervain’s tenosynovitis.

Tendonitis of the flexor carpi radialis is due to repetitive wrist flexion or acute overstretching of the wrist as can be seen in volleyball or water polo [20]. Pain develops from tendon thickening as it runs in its tunnel adjacent to the carpal tunnel. Pain typically courses from the radial palmar wrist crease towards the base of the second metacarpal made worse by resisted wrist flexion.

Conservative treatment for these tendinopathies begins by avoiding inciting events. Immobilization, stretching techniques, ice, and nonsteroidal anti-inflammatory medications can effectively diminish symptoms. Should symptoms persist, anesthetic/corticosteroid injections into the responsible tendon sheaths at the point of maximal tenderness can be of diagnostic and of therapeutic benefit. When recalcitrant to conservative measures, surgical release of the respective tunnel or compartment may be warranted.

### Ulnar-sided wrist injuries

#### Extensor carpi ulnaris injury

Abnormalities of the extensor carpi ulnaris (ECU) covers an array of pathologies seen in golf, baseball, hockey, tennis players, and other racquet sports. Injury may present as acute or chronic encompassing tendinosis, subluxation, dislocation, or rupture causing pain with or without mechanical symptoms on the ulnar side of the wrist. The pathophysiology involves repetitive microtrauma or a sudden traumatic episode during wrist flexion, supination, and ulnar deviation such as the nondominant hand in a double-handed backhand in tennis or the leading hand in the downward phase of a golf stroke.

Injury to the ECU will typically present with pain over the ulnar aspect of the wrist. Tenderness to palpation in the ECU groove and pain with resisted extension and ulnar deviation are hallmarks of tendinopathy. Subluxation will give the sensation of snapping with supination and ulnar deviation of the wrist. The physician should also evaluate the triangular fibrocartilage complex (TFCC) as a peripheral tear can lead to ECU tendonitis. Radiographic assessment is not routinely required unless needed to rule out other causes of ulnar-sided wrist pain. Ultrasound (US) can be useful in identifying inflammatory changes or using a dynamic assessment to look for tendon subluxation or dislocation [21]. MRI can be helpful to assess other structures such as the TFCC.

**Fig. 3:**
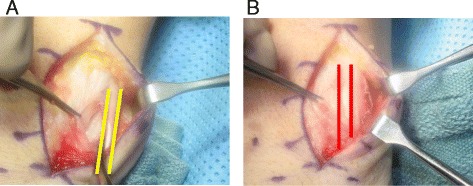
**a** Intraoperative finding of a volarly subluxed ECU tendon (*between yellow lines*) in a recreational tennis player. **b** The ECU tendon back in its reduced position (*red lines*) after an anatomic repair of the subsheath

Acute or chronic ECU tendinopathy can be managed with immobilization in wrist extension and ulnar deviation with progression to isometric and eccentric exercises. In cases of acute dislocation, reduction and immobilization with the forearm in pronation and the wrist in radial deviation for 4 months can be successful but not conducive to athletic participation [22]. Nonanatomic reconstruction of the subsheath with extensor retinaculum [23, 24] or, preferably, anatomic repair (Fig. 3) with reduction of the periosteum and subsheath back in the ulnar groove [25] are successful options to return to sports.

#### Ulnar abutment

The majority of load absorbed at the wrist is through the radiocarpal joint. In the ulnar neutral wrist, the distal ulna bears approximately 20 % of forces. As the wrist becomes more ulnar positive, the ulnocarpal joint experiences increased forces leading to ulnar-sided wrist pain. Ulnar positivity can be a normal anatomic variant, the result of distal radius physeal arrest (so-called gymnast’s wrist), or as a dynamic condition with grip and pronation [26, 27].

Rarely presenting after an acute injury, symptoms from ulnar abutment typically manifest as an insidious onset of pain with repetitive activities of pronation, gripping, ulnar deviation, axial loading, or combinations thereof which begin to affect the athlete’s level of play. Tenderness to palpation at the prestyloid recess of the ulna and pain with wrist ulnar deviation as moved through a full arc of pronosupination (ulnocarpal stress test) [28] is characteristic of the exam. Standard PA radiograph may reveal ulnar positivity but when dynamic ulnar positivity is suspected, a pronated/maximum grip PA view may be helpful in making the diagnosis [27]. MRI is not always necessary but may be helpful in evaluating the TFCC, early chondral changes in the distal ulna and/or the ulnar lunate, or lunotriquetral interosseous ligament tears.

**Fig. 4:**
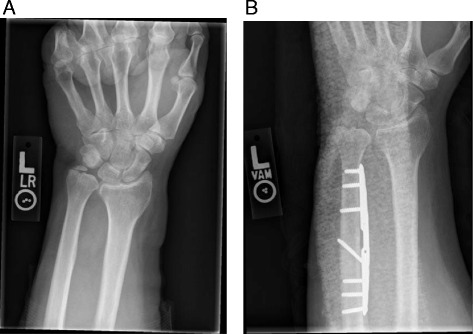
**a** PA radiograph of a patient with ulnar abutment revealing both 6 mm of ulnar-positive variance and incidentally an ulnar styloid nonunion. **b** Neutral to −1 mm of ulnar variance after a diaphyseal ulnar shortening osteotomy is performed

As a slowly progressive condition, acute surgical treatment is rarely warranted. Conservative measures to decrease symptoms and avoid provocative activities can allow continued participation. Immobilization between practices with or without nonsteroidal anti-inflammatories can decrease pain. Corticosteroid injections as a diagnostic and therapeutic tool can be used in the more chronic setting [29]. If conservative measures fail to allow continued level of play or timing is optimal, surgical treatment can be used to halt the progression by decreasing ulnar positivity and debriding the degenerative TFCC tear. Arthroscopic debridement and ulnar shortening are the mainstays of treatment. While arthroscopic wafer resection enjoys the benefit of shorter recovery times [30–32], the gold standard is diaphyseal ulnar shortening osteotomy (Fig. 4) and has shown improvement in pain, motion, and function [33–35].

#### Triangular fibrocartilage complex tears

Another cause of ulnar-sided wrist pain, particularly in those athletes who grip and rotate baseball bats, racquets, or golf clubs, is injury to the TFCC. The TFCC is a soft tissue complex that supports the distal radioulnar joint. It also acts as an extension of the radial articular surface serving as a load-bearing structure of the carpus on the distal ulna [3, 36]. In the acute setting, tears of the TFCC can occur with hyperextension and pronation of the axially loaded, ulnar deviated wrist. However, micro- or repetitive trauma can cause peripheral tears to the TFCC with rapid supination-pronation of the ulnar deviated wrist as seen with swinging a baseball bat.

**Fig. 5:**
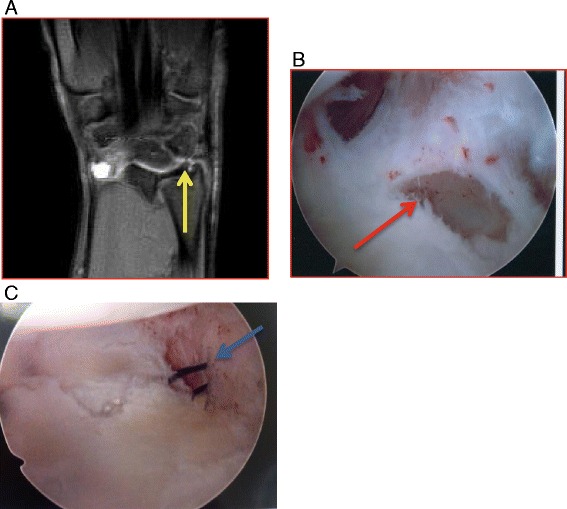
**a** A T2-weighted coronal sequence of a wrist MRI revealing an ulnar-sided peripheral tear of the TFCC (*yellow arrow*). **b** Arthroscopy view from the three to four portal showing the peripheral tear (*red arrow*). **c** Intraoperative arthrosopic image during an arthroscopic-assisted outside-in repair using PDS suture (*blue arrow*)

Deep aching pain, pain with gripping, and occasionally mechanical symptoms of clicking with pronation-supination can be experienced. Tenderness at the prestyloid recess that is accentuated with extremes of rotation or translation of the ulna, anterior to posterior, is characteristic of the exam. Standard radiographic evaluation typically appears normal. MRI or MRA are commonly used to confirm the diagnosis (Fig. 5a) [37].

As repetitive trauma is more common in athletes, conservative treatment is typically employed if symptoms arise during season. Immobilization, with or without physical therapy if ECU irritation is involved, for a period of 3 months can be helpful at alleviating symptoms [38]. Recalcitrant or recurring symptoms require arthroscopy for definitive classification as set forth by Palmer [36]. Symptomatic peripheral TFCC tears should be repaired either open or with arthroscopic assistance (Fig. 5b, c) [39–43] and typically require 3 months before return to play. Symptomatic tears of the central articular disk which fail conservative management can be treated with arthroscopic debridement (with or without a concomitant ulnar shortening osteotomy if indicated), but are not amenable to repair.

#### Hook of the hamate fractures

Direct blows from a golf club with the ground or from a baseball bat while “checking” a swing can result in hook of the hamate fractures. Infrequently, repeated lesser impacts from the same can result in stress fractures.

**Fig. 6:**
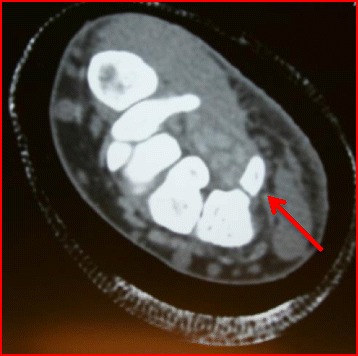
Axial CT image demonstrating a hook of the hamate fracture (*red arrow*) in a college hockey player

Hypothenar pain is present with palpation or with forceful grip. A pull test is performed by flexing the ring and small finger in the ulnar deviated wrist which produces pain by the deforming force of the flexors. Because the hook makes up one border of Guyon’s canal, dysesthesias in the ulnar nerve distribution or weak grip may be present. A carpal tunnel radiograph, in addition to standard PA and lateral views, is needed to make an accurate diagnosis. If radiographs are negative, a CT scan can be most helpful in defining the bony injury (Fig. 6).

Most fractures on presentation are subacute or chronic making definitive treatment with immobilization difficult. Whalen et. al. [44] reported healing of all six fractures they treated with immobilization, but other reports have showed less success and may risk flexor digitorum profundus (FDP) tendon injury [45, 46]. Biomechanical studies have suggested a possible decrease in flexion force with hook of the hamate excision lending consideration for open reduction and internal fixation [47, 48]. Nonetheless, excision of the hook of the hamate fragment is currently the standard of care and has produced successful results with return to play in 6 weeks [49–53].

### Hand/finger injuries

#### Thumb ulnar collateral ligament tears

Ulnar collateral ligament (UCL) injuries of the thumb are extremely common [54, 55] and often seen in skiing, basketball, and football. Injury occurs from an abduction moment at the thumb metacarpophalangeal joint (MCPJ) such as a fall onto an outstretched hand with the thumb abducted. An acute thumb UCL injury has been dubbed a skier’s thumb [56], in contrast to chronic attritional insufficiency of the ligament which is referred to as a gamekeeper’s thumb [57].

**Fig. 7:**
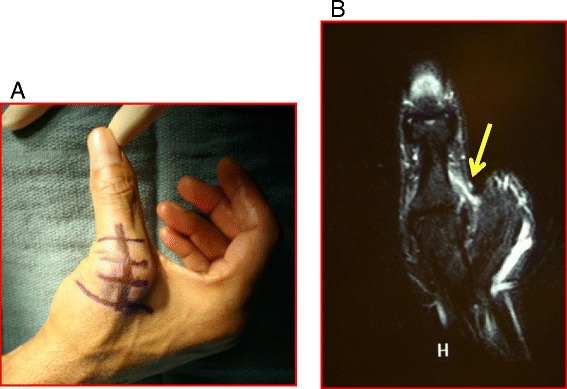
**a** Preoperative photograph demonstrating a patient with no endpoint to valgus stress testing of the thumb MCP joint. **b** A T2-weighted coronal sequence demonstrating a complete tear of the UCL which is detached from the proximal phalanx (*yellow arrow*)

Acute injuries often present with pain, ecchymosis, and swelling on the ulnar aspect of the thumb MCPJ. Stress examination with the thumb in extension and 30° of flexion is the most important aspect of the physical exam [58]. Laxity of 30° total, greater than 15° to the contralateral, or lack of endpoint (Fig. 7a) are all strongly suggestive of ligament injury [59, 60]. The thumb UCL has two portions, the proper (more dorsally located) and the accessory (more volar) ligaments. Laxity at 30° of MCPJ flexion and at full MCPJ extension is suggestive of injury to both the proper and the accessory components, respectively. Radiographic assessment is important for excluding bony fragments but US or MRI (Fig. 7b) is often used to confirm the diagnosis. A Stener lesion refers to interposition of the adductor aponeurosis in between the torn off UCL and its proximal phalanx insertion, making healing impossible.

Immobilization with a hand-based thumb spica splint or a cast with the interphalangeal (IP) joint free is appropriate for treating UCL partial tears with a firm endpoint to valgus stress testing at the MCPJ. For complete tears without an endpoint, surgery is recommended. Most UCL injuries are amenable to direct repair using either transosseous sutures or a suture anchor, although more chronic tears may require reconstruction with a variety techniques available [61–63]. Both UCL repair and reconstruction have shown satisfactory results with decreased pain and increased function [64].

#### Metacarpal/phalangeal fractures

Accounting for 10 % of all fractures presenting to the emergency department, metacarpal and phalangeal fractures are common injuries [65–67]. Injuries occur from falls, direct blows, or crush during sporting activity, although stress fractures have rarely been noted in racquet sports [68, 69]. Incidence is highest in contact sports such as football, lacrosse, and hockey [2, 70–72].

While swelling, ecchymosis, and deformity can be present, not all fractures lead to obvious deformity. For those with obvious deformity, a reduction maneuver should not be attempted without radiographic or fluoroscopic examination first in order to ensure appropriate treatment of the specific fracture, dislocation, or fracture dislocation [73]. In less obvious injuries, careful clinical examination of the hand with respect to digital range of motion (ROM), the finger cascade, and comparison of any subtle malrotation to the contralateral hand might point to an occult injury. Radiographic assessment with anteroposterior (AP), oblique, and lateral views are standard. Training rooms have been increasingly outfitted with mini-fluoroscopy units for rapid evaluation, although their sensitivity in detecting fractures of the smaller bones with possible intra-articular involvement has been questioned [74]. If further imaging for fracture characteristics is needed, a CT scan may be indicated.

Many fractures can be treated nonoperatively if acceptable alignment can be maintained with immobilization. When conservative treatment is inadequate, operative fixation is indicated. In the athlete, operative fixation may be sought to allow faster return to play.

#### Metacarpal fractures

Metacarpal base fractures occur from an axial load with the wrist in flexion. Eponyms such as Bennett and reverse-Bennett fractures are used to describe the characteristic fractures of the thumb and small finger metacarpal. Bennett fractures are sometimes associated with significant displacement as strong muscular forces tend to pull the base of shaft in abduction and proximally. As an intra-articular fracture, acceptable alignment to decrease the chance of symptomatic, posttraumatic arthritis is desirable [75]. If there is more than 25 % articular involvement or more than 1 mm of articular step-off or gapping between fracture fragments, operative fixation is usually indicated. Closed or open reduction of the fracture stabilized with Kirschner wires (K-wires) or screws is frequently needed.

**Fig. 8:**
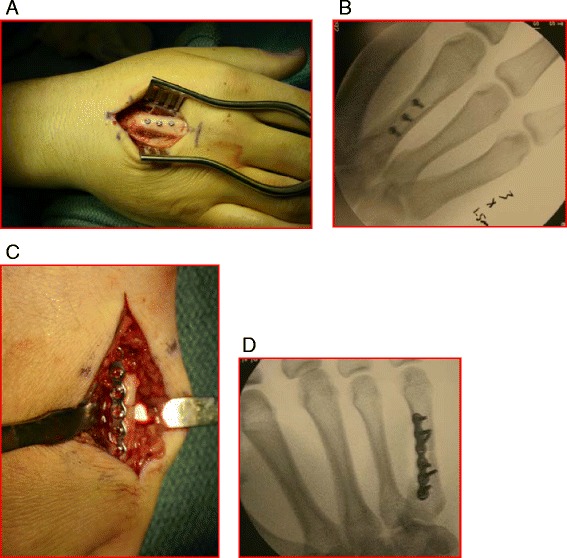
**a** Intraoperative and **b** fluoroscopic images of a long oblique metacarpal shaft fracture secured with three lag screws. **c** Intraoperative and **d** fluoroscopic images of a transverse shaft fracture secured with a plate and screws

Metacarpal shaft fractures are typically stable due to the intermetacarpal ligaments, although the net flexion moment at the distal segment pulls these fractures into a classic apex dorsal position. Acceptable angulation depends on the metacarpal involved with no greater than 10° tolerated in the index and up to 30° in the small finger [76]. Shortening of greater than 2 mm is generally not well tolerated as it leads to an extensor lag that can eventually not be compensated for by the hyperextensible MCPJ [77]. Careful clinical exam should assess not only for the finger cascade but also for the rotational deformity. Mild rotation in the metacarpal can lead to significant finger overlap [76]. Immobilization of isolated fractures in acceptable alignment is usually possible but the type of sports and position can limit tolerability. Multiple forms of fixation are possible each with its own relative advantages and disadvantages. K-wire fixation offers a soft tissue friendly form of fixation, adequate in maintaining alignment but protruding pins risk infection and pin migration/breakage and preclude further participation with exposed hardware in gripping athletics (i.e., tennis, basketball, golf). Lag screw fixation (Fig. 8a, b), indicated in long oblique fractures, offers minimal dissection and an anatomic reduction, but stability may not allow an expedited return to play. Plate and screw fixation (Fig. 8c, d) offers the stability of a relatively quicker return to play [78], but may expose the player to an increased risk of infection, tendon irritation, extensor adhesions, and the need for future hardware removal. Which treatment is selected should be a collaboration between the surgeon, the athlete, and the training staff.

**Fig. 9:**
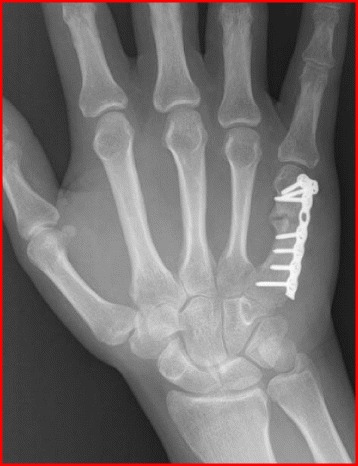
Plate and screw fixation of an angulated fifth metacarpal neck fracture in a high school running back

Metacarpal neck fractures are the most common metacarpal fracture as they occur at the metadiaphyseal junction at the area of the weakest bone. The so-called Boxer’s fracture is an eponym referring to metacarpal neck fractures of the small finger which result from an impact punching with a closed fist. Immobilization is typically adequate. Various forms of immobilization with the hand in intrinsic plus position or buddy taping with a short arm cast do not show any functional difference in outcome [79]. Acceptable alignment follows the same principles as for metacarpal shaft fractures with apex dorsal angulation being the most obvious deformity and increasing in toleration as the injury moves from the index to the small finger, with approximately 40°–50° of apex dorsal angulation well tolerated in the small finger. When acceptable alignment cannot be achieved or immobilization cannot be tolerated for the athlete, operative fixation is occasionally considered. Both K-wire and plate (Fig. 9) fixation have produced reasonably good outcomes, each with its own inherent risk/benefit profile as previously discussed.

#### Phalangeal fractures

**Fig. 10:**
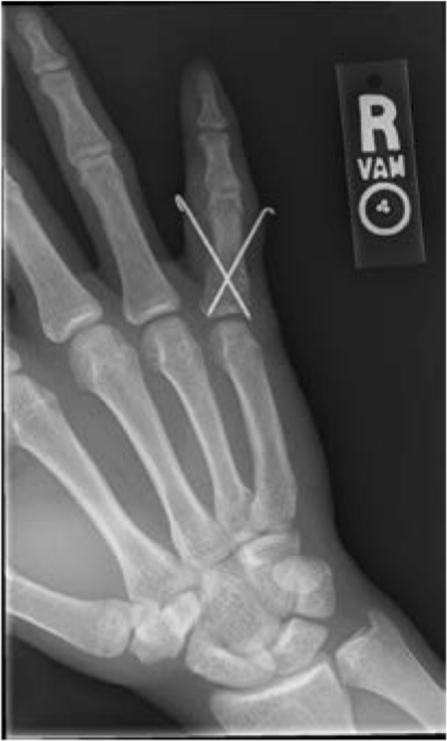
A PA radiograph of a small finger transverse proximal phalanx fracture with clinical malrotation which was treated with closed reduction and crossed K-wires

Shaft fractures of the proximal and middle phalanges can occur in a variety of patterns, but buddy taping and/or protective splint wear in acceptable alignment can allow fast return to play. Extra-articular fractures without rotational malalignment, less than 15°of angulation, and less than 6 mm of shortening are indicated for conservative treatment [80]. Operative fixation with open versus closed reduction using either K-wires (Fig. 10), screws, or plate and screws as fixation is sometimes required, especially when there is digital malrotation [81]. The athlete’s demands, the status of the season, and the fracture’s characteristics combine to dictate the optimal form of treatment.

When fractures enter the articular surface of the phalangeal base or condyle, operative fixation should be sought unless less than 1 mm of gap or step-off is present. Fractures can range from simple articular fractures, fixed with K-wires or screws, to comminuted pilon-type fractures which may require a distraction fixator in order to restore articular alignment through ligamentotaxis [82–84].

Distal phalanx fractures, due to crush mechanisms, are typically stable with surrounding soft tissue and the overlying nail plate. The vast majority of these are treated nonoperatively; however, careful attention should be paid to those distal phalanx fractures with associated nail bed trauma, such as displaced physeal (Seymour type) fractures in children.

**Fig. 11:**
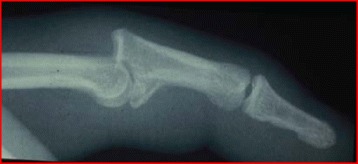
Lateral radiograph of a dorsal fracture dislocation of the PIP joint with 50 % articular involvement of the middle phalanx

Dislocations or fracture dislocations, particularly those that spontaneously reduce on the field, can often be overlooked in athletics. These comprise a spectrum of hyperextension jamming-type injuries from pure dorsal proximal interphalangeal (PIP) joint dislocations, to dorsal dislocations with volar plate avulsion fractures, to fracture dislocations involving a significant portion of the middle phalangeal articular surface. Radiographic assessment should be performed after every apparent dislocation to assess the percentage of articular surface involvement. In the setting of an acute PIP dislocation, with or without a volar plate avulsion fleck of bone, the PIP joint is most likely stable and early flexion ROM rehabilitation with buddy taping is appropriate. Fracture dislocations of the PIP joint which involve more than 30 % of the middle phalangeal articular surface may be unstable and should be treated much more cautiously, often requiring surgery. Those PIP fractures which involve more than 50 % of the PIP joint surface (Fig. 11) are clearly unstable and require surgical management.

#### Sagittal band rupture

A Boxer’s knuckle refers to an injury of the sagittal band, which is the structure that normally keeps the extensor digitorum communis (EDC) tendon centralized over the metacarpal head at the level of the MCP joint [85]. The sagittal bands are composed of transverse, sagittal, and oblique fibers that can be injured by blunt trauma over the MCPJ with a clenched fist impact [86, 87]. Painful EDC tendon subluxation can ensue causing an inability to achieve active extension of the finger at the MCP joint (cannot obtain, but can maintain extension).

Athletes can present with an acute or chronic injury. The central rays are more often affected due to more prominent bony structure, thinner superficial tissue, longer radial fibers, and single extensor tendons [86, 88]. Weakness of MCPJ extension in the affected digit, painful tendon subluxation usually in an ulnar direction, and tenderness over the injured sagittal band are evident on exam.

**Fig. 12:**
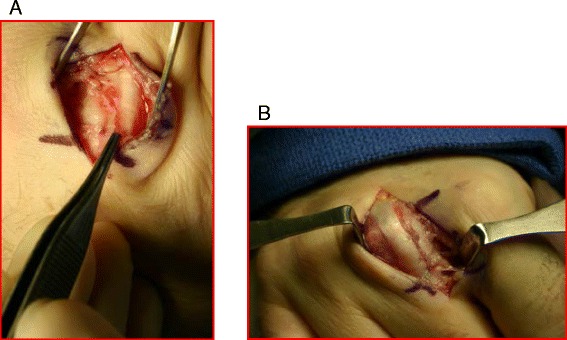
**a** Intraoperative findings of a patient with a torn radial sagittal band and ulnarly subluxed EDC tendon before repair **b** and after repair

Sagittal band injury without subluxation or dislocation can be treated with MCPJ extension splinting with the PIP joint free. When there is frank EDC subluxation or dislocation, a trial of conservative treatment can still be attempted, although results in the literature have been mixed [89–91] which have lead most surgeons to treat these injuries operatively (Fig. 12b, c) [87, 92]. MCPJ immobilization after repair or reconstruction is required to allow adequate healing after which aggressive ROM can be initiated. Athletes should be cautioned about returning to sports too quickly to prevent wound complications and recurrence [91, 92].

#### Central slip ruptures

Volar dislocation or forced flexion at the PIP joint can lead to acute rupture or chronic attenuation of the triangular ligament at the distal end of the central slip. These injuries are seen more commonly in basketball and volleyball players [93]. Injury leads to the lateral bands migrating volarly with resultant PIP joint flexion and hyperextension at the distal interphalangeal (DIP) joint known as a boutonniere deformity. As chronicity sets in, progressive loss of motion is seen at the PIP and DIP joints.

Evaluation with history and physical examination should elicit any history of volar PIP joint dislocation and try to isolate pain to the central slip insertion. Assessing DIP flexibility with the PIP joint resisted in extension (Elson’s test) is a helpful method for assessing the central slip [94]. An intact central slip would have a flexible DIP while an incompetent central slip would have a rigid DIP. Radiographs should be obtained with or without a history of dislocation to assess for bony avulsion of the central slip and PIP joint alignment.

Splinting of the affected digit with the PIP joint in extension and PIP free is appropriate in order to allow the central slip tendon to heal in as closed to an anatomic position as possible. Leaving the DIP joint free for flexion assists in pulling the lateral bands into normal alignment and decreases stiffness [95]. Athletes close to season completion can be allowed to continue competition as long as their participation is not hindered by the splint [96]. Rarely do acute injuries require operative treatment unless a displaced bony fragment is identified and requires screw fixation versus excision with repair [97] after which early rehabilitation can begin [97, 98].

Chronic central slip injuries with a fixed Boutonniere deformity create a challenging situation for the treating surgeon. Treatment begins by attempting to obtain a passively correctable deformity by using an extension splint, serial casting, or a dynamic external fixator [99–101] to stretch the contracted volar structures. Once a supple deformity is achieved, reconstruction can be attempted with a variety of techniques such as extensor tenolysis and transverse retinacular ligament mobilization or release, terminal extensor tenotomy with lateral band lengthening, and central slip reconstruction [102–104]. Because treatment of a chronic deformity results in much worse outcomes [97, 105, 106], athletes should be strongly encouraged to seek treatment in the acute period.

#### Pulley ruptures

Closed annular pulley ruptures occur most commonly in rock climbers due to the high demand placed on the flexor tendon system in the hanging and crimping positions [107, 108]. Pulley ruptures typically involve the A2 or A4 pulleys and occur most often in the middle and ring fingers. Previous work has evaluated the force required to produce an A2 pulley tear and loads experienced during these vulnerable maneuvers finding they are at particular risk for climbers [109–112].

Athletes present with the acute onset of pain over the volar aspect of the affected digit which may exhibit swelling and ecchymosis. They can usually isolate the event to a particular movement or slip leading to a forceful digital contraction and feeling a pop. Tenderness to palpation can usually be localized over the affected pulley but diffuse swelling of the entire flexor tendon sheath may cause pain with passive extension. While rupture of the A2 and A4 pulley are generally required to show significant bowstringing, relative bowstringing may be appreciated or a flexion lag may be evident on exam. Applying external pressure over the affected area and asking the patient to flex the digit may reduce his or her pain further supporting the diagnosis. While pulley ruptures are not evident on plain radiographs, MRI or US may be helpful in confirming the diagnosis [113–116].

Isolated pulley ruptures can be effectively treated nonoperatively with taping or pulley rings that externally provide support for the flexor tendon. However, in the case of multiple pulley ruptures, or failed nonoperative treatment, reconstruction is indicated [117]. A variety of graft sources such as palmaris longus tendon, extensor retinaculum, or an excised flexor digitorum superficialis (FDS) slip are available. Early ROM to facilitate tendon gliding is encouraged with higher loading not allowed until 6 months postoperatively.

#### Jersey finger

Forceful hyperextension of the DIP joint leading to FDP avulsion, as seen with a jersey tearing away from a finger, is most commonly seen in football and rugby players. Eccentric loading of the FDP has shown the ring finger to be most susceptible to injury due to its position in power grip, decreased independent motion, and failure strength compared to other digits [118–122].

**Fig. 13:**
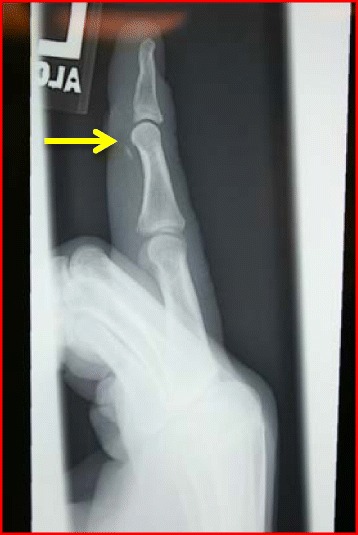
Lateral radiograph of a ring finger of a patient whose finger got caught on a basketball net showing an FDP bony avulsion fracture with the fragment caught up distal to the A4 pulley level (*yellow arrow*)

The athlete may or may not recall the moment of injury to the digit. Swelling is commonly present which may cause the athlete to not seek attention assuming that the lack of DIP flexion is simply a fingertip sprain. Commonly, they present with complaints of decreased motion or stiffness and lack of strength. Continuity of the flexor system can be assessed passively by tenodesis effect or by holding the MCPJ and PIP joints extended and asking the athlete to flex the DIP in isolation. In cases of a bony avulsion (Fig. 13) in which the trapped fragment cannot migrate proximally through the A4 or A5 pulley, some flexion may actually be possible but decreased and painful. Radiographic assessment can be helpful at identifying a bony avulsion with amount of retraction. For pure tendon failures, MRI can provide information about continuity and retraction of the tendon [123, 124], but is not usually necessary.

Further migration or retraction of the tendon can compromise the nutritional supply to the flexor tendon. Therefore, operative intervention is warranted as soon as possible. Various methods for repair have been described, but they all involve advancement of the intact, viable tendon to the base of the distal phalanx, often using transosseous sutures tied over a dorsal button or suture anchors [125–129]. Chronic injuries may require primary or staged flexor tendon reconstruction with a tendon graft, or in the case of an intact FDS, a DIP joint arthrodesis could be considered [121].

#### Mallet finger

Mallet finger injuries refer to the disruption of the terminal extensor tendon from the distal phalanx, with or without an avulsed bony fragment. Its occurrence most commonly in baseball has lead to the eponym “baseball finger” [130], but is also seen in football, basketball, and rugby [131]. Its mechanism of injury involves forceful flexion of an extended DIP joint.

**Fig. 14:**
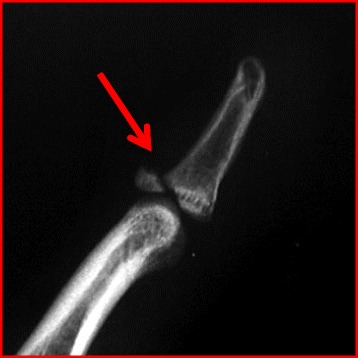
Lateral radiograph of a mallet finger with a bony avulsion fragment (*red arrow*)

The hallmark of physical exam for mallet fingers is a fingertip that is “drooped” in flexion with the inability to extend at the DIP joint. Dorsal DIP swelling and ecchymosis are commonly seen but in cases without bony involvement are often surprisingly painless [132]. Evaluation should include assessment for swan neck deformity (flexed DIP with extended PIP) as this may cause more functional deficit than a DIP flexion deformity. Radiographic assessment is necessary to assess for a bony mallet avulsion fragment (Fig. 14) and alignment of the DIP joint.

Conservative treatment with extension splinting of the DIP joint is appropriate for almost all mallet fingers, including those with bony fragments as long as there is no significant joint subluxation [133–135]. Full-time DIP splinting with the PIP joint free is recommended for 6 weeks around the clock oftentimes with an additional 6 weeks of nighttime splinting [136–141]. For athletes experiencing splint complications such as dorsal skin maceration or difficulty with the compliance of full-time splinting, buried K-wire immobilization of the DIP joint offers an alternative treatment path with possible return to sports albeit with relatively high inherent risks [142].

## Conclusions

Hand and wrist injuries in athletics are common and can have a significant impact in multiple areas. Knowledge of these entities and special consideration for the athlete can help the team physician effectively treat these players and help them achieve their goals.

## Abbreviations

PA: Posteroanterior
CT: Computed tomography
MRI: Magnetic resonance imaging
SL: Scapholunate
DISI: Dorsal intercalated segmental instability
ECU: Extensor carpi ulnaris
TFCC: Triangular fibrocartilage complex
US: Ultrasound
FDP: Flexor digitorum profundus
UCL: Ulnar collateral ligament
MCPJ: Metacarpophalnageal joint
IP: Interphalangeal
ROM: Range of motion
AP: Anteroposterior
K-wires: Kirschner wires
PIP: Proximal interphalangeal
EDC: extensor digitorum communis
DIP: Distal interphalangeal
FDS: Flexor digitorum superficialis
